# Cavernous lymphangioma of the breast

**DOI:** 10.1186/1477-7819-5-69

**Published:** 2007-06-20

**Authors:** Gabriel O Ogun, O Oyetunde, EffiongE Akang

**Affiliations:** 1 Department of Pathology, University College Hospital, PMB 5116, Ibadan, Nigeria; 2 Department of Surgery, Jericho General Hospital, Ibadan, Nigeria

## Abstract

**Background:**

Cavernous lymphangioma is a rare lesion in the breast of adults. Only a few cases have been documented in literature.

**Case presentation:**

We describe a 38-year-old woman who presented with a palpable breast lump, which measured 5 × 4 cm. A local excision of the lump was performed and a diagnosis of cavernous lymphangioma was made. The patient is alive and well, after five years of follow-up, with no complaints or recurrence.

**Conclusion:**

To the best of our knowledge, this is the first case to be documented in a black African woman. Complete surgical excision seems to be the best modality of treatment of this lesion.

## Background

Lymphangioma in the breast is a rare entity, and only a few cases have been documented in the literature [1-9]. Lymphangiomas are composed of dilated lymphatic channels lined by endothelium. They occur predominantly in children, with up to 90% of cases presenting by the second year of life [10,11]. The cases that have been previously documented in literature ranged from three to 25 cm in diameter and were mainly located in the upper outer quadrant of the breast (Table 1). This anatomical pattern of distribution is related to the drainage pattern of the lymphatics in the breast, which is mainly towards the tail and the axilla [9]. We present a case of lymphangioma in the breast of a black African woman.

**Table 1:** Clinical details of documented cases of cystic lymphangioma of the breast

**Authors**	**Age in years**	**Sex**	**Size (cm)**	**Location of lump in the breast**	**Presenting symptoms**	**Duration of presenting symptoms**	**Management**	**Follow up duration**
Torscasio *et al *[1]	26	F	4 × 3 × 2	Inner quadrant	Lump, no pain	2 months	Surgery alone	24 months with no recurrence
de Guerké *et al *[2]	31	F	3	Upper outer quadrant	Lump	Not stated	Conservative	Patient was pregnant at diagnosis, and was followed up for 8 weeks postpartum
Sa *et al *[3]	36	F	3.5 × 3	Upper outer quadrant	None	Detected on routine mammography	Surgery alone	Not stated
Kangesu [4]	6	M	6 × 5	The whole breast	Lump with discoloration of overlying skin	Since birth	Surgery alone	Not stated
Sieber *et al *[5]	49	F	7 × 7 × 2	Upper outer quadrant	Lump	30 months	Surgery alone	Not stated
Kurosumi *et al *[6]	16	F	16 × 14	Not stated	Lump	24 months	Surgery alone	Not stated
Chiba *et al *[7]	4 months	M		The whole breast	Lump	Since birth	Surgery alone	Not stated
Waqar *et al *[8]	24	F	25 × 20 × 7	The whole breast	Lump, progressive increase in size, pain	Since birth	Surgery alone	Not stated
Chung *et al *[9]	34	F	10	Upper outer quadrant	Lump, no pain	Not stated	Surgery alone	Not stated
Ogun *et al *(present case)	38	F	5 × 3 × 2.5	Upper outer quadrant	Lump, pain	2 months	Surgery alone	60 months with no recurrence

## Case presentation

A 38-year-old black female presented with a two-month history of a painful left breast lump. The pain was relieved by simple analgesics. There was no associated nipple discharge. There were no other clinical symptoms. Her past medical and family history was not significant. Physical examination revealed a well circumscribed, slightly mobile, and tender soft lump measuring about 5 × 4 cm, located in the upper outer quadrant of the left breast. There was no axillary lymphadenopathy. Clinical examination did not reveal any other significant findings. A fine needle aspiration cytology specimen obtained from the breast lesion was reported as inflammatory. The patient subsequently had excisional biopsy of the lump.

**Figure 1:**
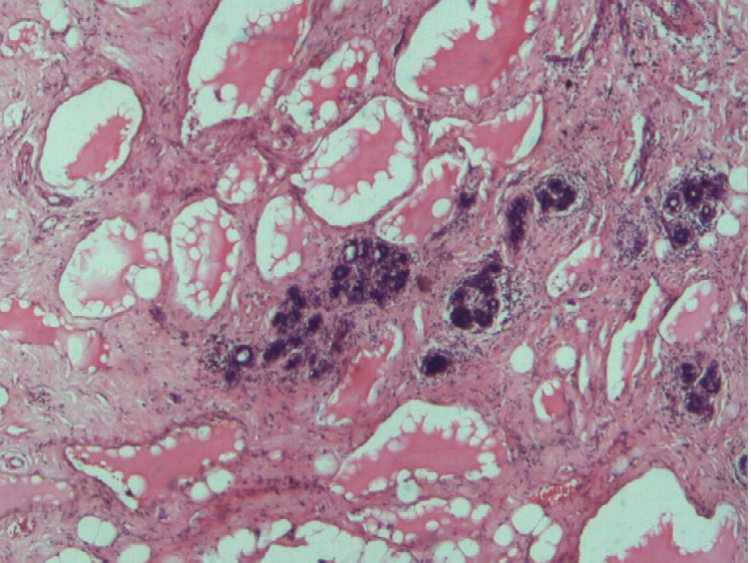
Distended lymphatic channels interspersed by breast lobules. (H & E ×40).

**Figure 2:**
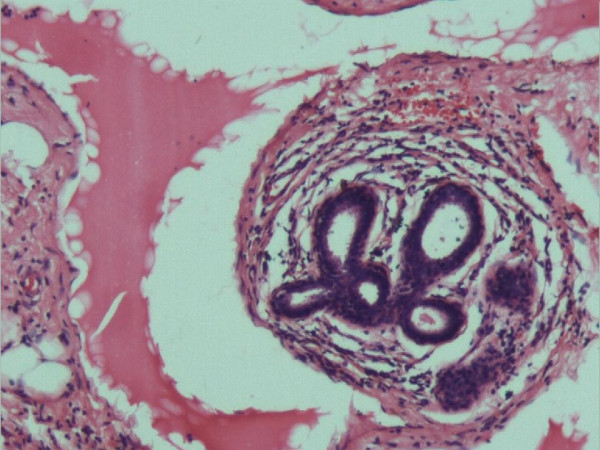
Higher power view of the lesion showing attenuated endothelial lining of a lymphatic channel. (H & E ×100).

Macroscopically, the lump measured 5 × 3 × 2.5 cm and weighed 30 gm. It was nodular, and soft in consistency. Serial sections of the lump revealed greyish white surfaces, with multifocal dark brown coloured areas. Microscopically, the tumour was composed of numerous cavernous and cystically dilated spaces lined by a single layer of flattened endothelial cells, and containing brightly eosinophilic lymphatic fluid. The distended lymphatic channels were supported by a prominent fibrocollagenous stroma. There was infiltration of the stroma by lymphocytes. Islands of adipose tissue and normal breast lobules were observed around the dilated lymphatic channels (Figures 1 and 2). The margins of the resection were free of the lesion. The definitive histological diagnosis was cavernous lymphangioma. The patient was offered no other treatment. Five years later, the patient is alive and well without any complaint or recurrence.

## Discussion

Lymphangiomas are relatively uncommon lesions, more so in the breast, where they are very rare. Some authorities regard lymphangiomas to be true neoplasms that are capable of local aggressive behaviour, but overall they are benign [11].

By contrast, others believe that lymphangiomas are hamartomatous malformations that result from the failure of the lymphatic system to communicate with the venous system. Another closely related view is that lymphangiomas represent sequestrated lymphatic tissue that fails to communicate normally with the lymphatic system. According to these latter two hypotheses, abnormal rests of lymphatic tissue possess some capacity to proliferate and accumulate vast quantities of fluid, accounting for their cystic appearance [11].

Lymphangiomas are classified as capillary, cavernous, and cystic forms. The distinction between cavernous and cystic lymphangioma is often arbitrary, since both components may occur in the same lesion, thus raising the possibility that the so-called cystic lymphangioma is merely a long-standing cavernous lymphangioma in which the cavernous spaces are very large sized [5,6,8].

Table 1 show the clinical features of cases already documented in literature along with our index case. The localization of most cases to the upper outer quadrant of the breast is probably related to the route of drainage of the lymphatic system of the breast [9].

Typically, lymphangiomas are described as fairly well circumscribed and soft, as was observed in our index case [1,6,11]. Imaging techniques such as ultrasonography, mammography, and magnetic resonance imaging may be used to assess, make a clinical diagnosis and for follow-up [1-3,5-9].

The final diagnosis is based on a combination of clinical, radiological, and histopathological findings [1,2,6,9].

Histologically, cavernous lymphangiomas show numerous narrow spaces containing amorphous eosinophilic fluid and few lymphocytes and lined by a monolayer of attenuated endothelial cells [6,11]. Some of the channels may contain blood because of secondary haemorrhage, resulting in misdiagnosis of this lesion as cavernous haemangioma. However, the presence of large collections of lymphoid cells in the stroma, sometimes with lymphoid follicle formation, and the relatively greater irregularity of the lumina of the cavernous spaces, tilt the diagnosis in favour of lymphangioma [11,12].

Vascular endothelial markers such as factor VIII- associated antigen and CD 31 may be positive in lymphatic endothelium and are therefore not reliable in distinguishing between haemangiomas and lymphangiomas [6,11]. Laminin may be expressed in the discontinuous basal lamina of lymph vessels [6,12]. It is best expressed in blood vessels than lymphatic channels, therefore making a sharp distinction between the two structures difficult.

A possible differential diagnosis is lymphangiomyomatosis, which exclusively occurs in women and is localised to the lymphatics of the mediastinum, retroperitoneum and the pulmonary parenchyma. Lymphangiomyomatosis is characterised by a distinctive and very prominent smooth muscle proliferation in the affected lymphatics and the smooth muscle cells co-express muscle and melanocytic markers [11,12]. Another differential diagnosis is acquired lymphangiectasis, which occurs in patients who have had surgery and radiation therapy for malignancy. Morbidly obese individuals may also develop lymphangiectasis, due to the weight of large dependent folds of fat causing lymphatic obstruction. This may be more pronounced with previous surgery interrupting the lymphatics [11].

Local surgical excision is the best modality of treatment of this benign breast disease, as is the practice for lymphangiomas in other parts of the body, and as illustrated by this case and previously reported cases. The probability of recurrence is low if completely excised.

## Conclusion

To our knowledge, this is the first case to be documented in a black African woman. Complete surgical excision seems to be the best modality of treatment.

## Competing interests

The author(s) declare that they have no competing interests.

## Authors' contributions

OGO and AEE designed the study, performed the histological assessment and picture acquisition and drafted part of the manuscript. OO performed the surgery, carried out data acquisition and drafted part of the manuscript. All authors participated in the editing and have read and approved the final manuscript.
